# Effectiveness and cost-effectiveness of transmural collaborative care with consultation letter (TCCCL) and duloxetine for major depressive disorder (MDD) and (sub)chronic pain in collaboration with primary care: design of a randomized placebo-controlled multi-Centre trial: TCC:PAINDIP

**DOI:** 10.1186/1471-244X-13-147

**Published:** 2013-05-24

**Authors:** Eric W de Heer, Jack Dekker, Jonna F van Eck van der Sluijs, Aartjan TF Beekman, Harm WJ van Marwijk, Tjalling J Holwerda, Pierre M Bet, Joost Roth, Leona Hakkaart-Van Roijen, Lianne Ringoir, Fiona Kat, Christina M van der Feltz-Cornelis

**Affiliations:** 1 Netherlands Institute of Mental Health and Addiction (Trimbos-institute), Utrecht, The Netherlands; 2 Tilburg School of Behavioral and Social Sciences, Tranzo Department, University of Tilburg, Tilburg, The Netherlands; 3 TopClinical Centre for Body, Mind and Health, GGz Breburg, Tilburg, The Netherlands; 4 The EMGO Institute for health and care research (EMGO+), Amsterdam, The Netherlands; 5 Department of Psychiatry, VU University Medical Centre, Amsterdam, The Netherlands; 6 GGz inGeest, Mental Health Institute, Amsterdam, The Netherlands; 7 Department of General Practice, VU University Medical Centre, Amsterdam, The Netherlands; 8 Arkin, Mental Health Institute, Amsterdam, The Netherlands; 9 iMTA, Erasmus University, Rotterdam, The Netherlands; 10 Department of Clinical Pharmacology and Pharmacy, VU University Medical Centre, Amsterdam, The Netherlands; 11 Tilburg School of Behavioral and Social Sciences, Department of Medical Psychology, Tilburg University, Tilburg, The Netherlands; 12 Department of Clinical Psychology, VU University, Amsterdam, The Netherlands

**Keywords:** Depression, Pain, Duloxetine, Collaborative Care, Transmural, Primary Care

## Abstract

**Background:**

The comorbidity of pain and depression is associated with high disease burden for patients in terms of disability, wellbeing, and use of medical care. Patients with major and minor depression often present themselves with pain to a general practitioner and recognition of depression in such cases is low, but evolving. Also, physical symptoms, including pain, in major depressive disorder, predict a poorer response to treatment. A multi-faceted, patient-tailored treatment programme, like collaborative care, is promising. However, treatment of chronic pain conditions in depressive patients has, so far, received limited attention in research. Cost effectiveness of an integrated approach of pain in depressed patients has not been studied.

This article describes the aims and design of a study to evaluate effects and costs of collaborative care with the antidepressant duloxetine for patients with pain symptoms and a depressive disorder, compared to collaborative care with placebo and compared to duloxetine alone.

**Methods/Design:**

This study is a placebo controlled double blind, three armed randomized multi centre trial. Patients with (sub)chronic pain and a depressive disorder are randomized to either a) collaborative care with duloxetine, b) collaborative care with placebo or c) duloxetine alone. 189 completers are needed to attain sufficient power to show a clinically significant effect of 0.6 SD on the primary outcome measures (PHQ-9 score). Data on depression, anxiety, mental and physical health, medication adherence, medication tolerability, quality of life, patient-doctor relationship, coping, health resource use and productivity will be collected at baseline and after three, six, nine and twelve months.

In the collaborative care conditions a) and b), a care-manager provides Problem Solving Treatment and integrated symptom management guidance with a self-help manual, monitors depressive and pain symptoms, and refers patients to a physiotherapist for treatment according to a 'Graded Activity' protocol. A psychiatrist provides duloxetine or placebo and pain medication according to algorithms, and also monitors pain and depressive symptoms. In condition c), the psychiatrist prescribes duloxetine without collaborative care. After 12 weeks, the patient is referred back to the general practitioner with a consultation letter, with information for further treatment of the patient.

**Discussion:**

This study enables us to show the value of a closely monitored integrated treatment model above usual pharmacological treatment. Furthermore, a comparison with a placebo arm enables us to evaluate effectiveness of duloxetine in this population in a real life setting. Also, this study will provide evidence-based treatments and tools for their implementation in practice. This will facilitate generalization and implementation of results of this study. Moreover, patients included in this study are screened for pain symptoms, differentiating between nociceptive and neuropathic pain. Therefore, pain relief can be thoroughly evaluated.

**Trial registration:**

NTR1089

## Background

Patients with major depressive disorder (MDD) and co-morbid chronic pain now have a high risk of not receiving optimal care [1-4]. The burden of co-morbid pain to depression is high for patients in terms of disability, wellbeing, and use of medical care [5]. Complex, integrated collaborative care including active pain management is perhaps better for this population than antidepressants alone, but there is limited evidence. Once such integrated care is available and effective, the added value of an antidepressant over placebo is also under debate. In this trial we aim to evaluate to what extent depressive symptoms improve in patients with MDD with concomitant pain symptoms of both 6–12 weeks and ≥ 12 weeks duration [6]: a) with collaborative care versus an antidepressant alone, and b) with an antidepressant versus placebo within the collaborative care condition.

There is a strong correlation between pain and psychiatric distress reporting [7-11]. Physical symptoms, including several pain symptoms, increase the likelihood of a depressive disorder [12]. Also, many patients with pain symptoms experience a depression [5,13,14], and when the number of pain symptoms increases, the prevalence of depression increases as well [15,16]. Vice versa, when the severity of depression increases, so does the severity of pain complaints [10]. Furthermore, several studies indicate that pain symptoms are common in patients with MDD: the ARTIST trial reports a 69% rate of pain symptoms in primary care patients with depression [17]; in psychiatric clinics in Spain, almost 60% of patients with depression had pain complaints [18]; an international telephone survey in 18,980 patients with MDD showed that 43.4% presented themselves with chronic painful physical conditions [19], with headache, back pain, and limb pain being the most prevalent; the mean prevalence of pain was 65% in a meta analysis of 14 studies [5], and a US telephone survey reported a 65.6% prevalence of chronic pain in depressed persons (N = 5808) [20]. Although a strong correlation has been found between depression and pain, it is not clear if depression causes pain [8], but it is suggested that pain could be a symptom of depression [5,9,21]

Moreover, depression and painful physical symptoms increase costs by increased utilization of healthcare services [5,22-24]. A 2.8 and 4 fold expenditure elevation is reported in depressed patients with back pain and migraine, respectively [25]. These costs are the so-called direct medical costs. But most costs of mental disorders in general, and depression specifically, are indirect costs, as a consequence of productivity loss and absenteeism: More than 70% of the total costs of depression consists of these indirect costs [26].

Therefore, good detection and diagnosis of comorbid conditions is necessary in the first place to be able to treat those conditions adequately [9,27]. In patients for whom psychiatric consultation was asked by a General Practitioner because of Medically Unexplained Symptoms including pain, up to 86% unrecognized depressive and anxiety disorders were found by the consulting psychiatrist [28,29]. Depression is high on the list of possible diagnoses if a patient presents with multiple unexplained physical symptoms including general aches and pains [5,30]. However, even in case of recognition, simple treatment of the depression is not sufficient. Physical symptoms including pain in MDD are associated with treatment resistance and predict a poorer response to treatment [1-4,31]. Greater risk of relapse, suicide, and substance abuse have been reported [32,33]. Residual symptoms in MDD predict relapse; patients with residual symptoms relapsed 3 times as fast compared to those who were asymptomatic at remission [34]. Among patients with residual symptoms, > 90% had mild-to-moderate physical symptoms including pain [32]. Improvement in painful physical symptoms is associated with higher remission rates in MDD [35]. In a study in newly referred neurologic outpatients, pain occurred in 10-55% of several diagnostic groups; and comorbid MDD occurred in 10-30%. Pain as well as MDD symptoms persisted over a period of 12 months, and the authors stated that for remission of both, interventions were needed specifically addressing pain symptoms as well as depressive symptoms [36].

Treatment of chronic pain in depressed patients has been addressed [33], with a focus on pain treatment. Particularly for patients with both depression and pain, flexible, integrated, multi-faceted [37], patient-tailored methods of treatment are needed, in combination with improvement of adherence, i.e. disease-management programs such as collaborative care (CC) [38-40]. Two recent meta-analyses have indeed shown the effectiveness of such collaborative care approaches in primary care [41,42]. Little is also known about the added effect of an antidepressant such as duloxetine over placebo when collaborative care is available.

Collaborative care is successful in the treatment of depression, can vary in content and intensity [42] and combines various interventions for the treatment of disorders and includes active monitoring of symptoms, through the use of questionnaires that are used at each session with a professional. The contents of collaborative care consist of a diagnostic assessment of complaints, and contracting, to improve adherence. Also, a self-help manual, guided by a care-manager, can be part of collaborative care. This manual contains information about different symptoms and how to cope with them; the coping can be addressed in terms of emotional coping, cognitive coping and behavioral coping (e.g. tips for a healthy lifestyle which can decrease physical complaints; information on pleasurable activities which can be done with complaints) [43-46]. Next to this manual, a psychotherapy is offered in CC. Mostly, this is cognitive behavioral therapy or problem solving treatment (PST), both effective in the treatment of depression [47]. Next to psychotherapy, medication can be an element of a collaborative care approach, guided by a psychiatrist. Several studies indicate that tricyclic antidepressants (TCAs) are effective in the treatment if pain and depression coexist, as well as selective serotonin and noradrenalin reuptake inhibitors (SNRIs) such as duloxetine [48] and venlafaxine, that have been shown to alleviate pain and depressive symptoms [49-52]. However, in a meta-analyses of Spielmans (2008) the analgesic effect of Duloxetine has been questioned [53], which calls for establishing the effect size for concomitant pain in depressed patients by duloxetine. In case of comorbid physical complaints, a physiotherapist can also be part of a collaborative care approach. Because treatment in CC is dependent on the process of symptom reduction, systematic monitoring of the complaints is an important part of CC. Furthermore, as a systematic review showed that psychiatric consultation to primary care with a consultation letter is effective for somatoform disorders [54], adding the use of such a consultation letter to CC can be useful. General practitioners (GP's) see treatment of complex comorbid conditions such as the comorbid condition under study as challenging and tend to refer these patients to either mental health care or pain specialists. Because GP's in general find treatment of this patient group difficult, the benefits of a collaborative care approach may be substantial and a transmural collaborative care approach may be useful [38-42].

Recently, a trial explored efficacy of collaborative care for MDD and musculoskeletal pain [55] and another trial explored efficacy of collaborative care for chronic pain in the primary care setting [23], both with positive results, but in one study the collaborative intervention was more expensive than care as usual [56]. However, treatment of other chronic pain conditions in depressive patients has, so far, not received much attention. Also, cost effectiveness of an integrated approach of pain in depressed patients has not been studied yet.

### Definition of (sub)chronic pain in this trial, and the way its treatment is addressed in the collaborative care arm

According to the international Association for the study of Pain (IASP), pain is *an unpleasant sensory and emotional experience associated with actual or potential tissue damage, or described in terms of such damage*[57]*.*

Most pain originate from an outside stimulus (heat or a sting), but may also result from injury to sensory fibres (or even from damage to the central nervous system itself), and follows an ascending pathway to the brain [58,59]. Through nociceptors, a signal is transmitted via the peripheral nervous system to the dorsal horn in the spinal cord, and from there to the brain, where the perception of pain is constructed. The dorsal horn acts as a pathway for transmission of nociceptive information [58-60]. In the brain, the descending control systems are activated (possibly through the spinomesencephalic tract). Serotonin (5-HT) appears to play an important role in the descending pathway of pain [60].

Serotonin, norepinephrine, substance p, glutamate, NMDA and gamma-aminobutyric acid (GABA) play a role in pain processing in rats and mice [61-65] and in humans [46,66]. Increasing the availability of norepinephrine and 5-HT may promote pain inhibition centrally [62]. At the central level, pain can be modulated by attention [67] and by mood [68]. In chronic pain the relationship between trigger and pain is not clear anymore (or detected tissue damage cannot fully account for pain intensity) and the pain persists past the normal time of healing. Also, in chronic pain, gray matter degeneration occurs in the anterior cingulated cortex and prefrontal cortex as well as in the left parahippocampal cortex [69], brain areas that are also implicated in depressive disorder and that may diminish the ability of patients to learn [70,71], and thus to follow treatment properly. Furthermore, chronic pain is associated with sleep disturbances [72] and chronic stress causes dendritic regression and loss of dendritic spines in hippocampal neurons that is accompanied by deficits in synaptic plasticity and memory [73].

In this study, in the collaborative care treatment, specific attention is paid to the management of pain by proper pain medication and adherence to pain medication. For this purpose, the pain is classified as nociceptive pain or neuropathic pain by a questionnaire, the PAINDETECT [74] and treatment of pain will be applied based on which of these two sorts of pain a patient has. Nociception leads to pain, but the amount of pain experienced depends on integration in the cerebral rostral centers, the dorsal horn, and in the peripheral input. Neuropathic pain occurs without a damaging stimulus of outside and is associated with increased excitation and decreased inhibition of ascending pain pathways. Nociceptive pain may have a protective nature, at least in its acute form, but neuropathic pain is the result of nervous damage [75]. However, in case of chronic pain, fibre loss occurs in the spinal cord and in the peripheral nerve, due to chronic central inhibition [63,76-78]. In some cases, the pain can be mixed. An algorithm is used for administration of pain medication according to the classification.

Confusion exists in terminology in describing physical symptoms and pain associated with depression: They may be somatised symptoms of MDD, or so-called associated symptoms; Medically Unexplained Symptoms, that frequently occur together with MDD; Chronic painful physical conditions that are symptoms of a chronic illness, such as diabetic neuropathy; or chronic painful conditions resulting from centralisation of a formerly peripheral pain symptom.

In this trial, we aim to improve depressive symptoms of patients with MDD with concomitant subchronic or chronic pain symptoms of nociceptive, neuropathic, mixed or functional nature. As discerning nociceptive pain from neuropathic pain may have therapeutic consequences in the treatment of (sub)chronic pain [74], we address both these issues in this RCT. We define subchronic pain as pain with 6–12 weeks duration, and chronic pain as pain of ≥ 12 weeks duration.

This Randomized Controlled Trial aims to evaluate effectiveness and cost effectiveness of this transmural collaborative care model with Consultation Letter (TCCCL) in patients with MDD and concomitant (sub)chronic pain.

## Methods/Design

### Objectives

The primary objective of this randomized placebo-controlled trial is to establish effectiveness on severity of depression (measured by Patient Health Questionnaire (PHQ-9)) of a closely monitored integrated intervention (TCCCL + duloxetine) for concomitant depression and (sub)chronic pain compared to either TCCCL + placebo and compared to Duloxetine alone.

Secondary objectives of this trial are to establish cost effectiveness in terms of Quality of Adjusted Life Years (QALY) as measured by EuroQol-5 and SF-36 and costs measured by TIC-P; and to establish improvement on pain in terms of Brief Pain inventory (BPI).

### The study will evaluate the following hypothesis

#### Hypothesis

Combined treatment (TCCCL + Duloxetine) is more effective than mono-treatment (either TCCCL + placebo or Duloxetine alone) in depressive symptom reduction on the PHQ-9 as main outcome for patients with MDD and (sub) chronic pain.

#### Study design

**Figure 1:**
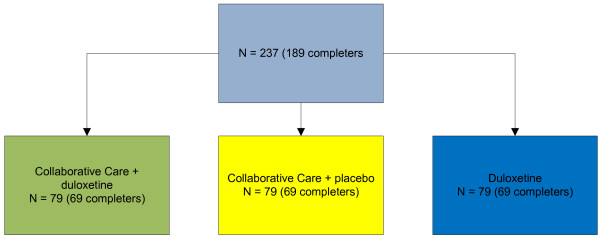
Treatment options.

This study is a three armed randomized Multi Centre trial led by GGz Breburg, with three collaborating mental health institutions: GGz Breburg, Arkin, and GGz inGeest, who will all include 73 patients in order to have 3 × 63 completers for the study. See Figure 1 for treatment options.

#### Participating mental health institutions

Three mental health institutions participate in this study. Patients will be treated at these institutions according to protocol. The participating mental health institutions are: GGz Breburg in Tilburg, GGZ inGeest in Amsterdam and Arkin/PuntP, also in Amsterdam.

#### Recruitment of care-managers and psychiatrists in the Mental Health Institutions

Care-managers and psychiatrists will be recruited at the three participating mental health centers (GGz Breburg, Arkin, GGz inGeest) and form treatment teams for the trial. The care-managers are psychiatric nurses or psychologists who receive training in care management (including PST) to provide in the collaborative care interventions. The psychiatrists are trained in prescribing duloxetine and placebo medications as well as the pain medication according to the algorithm and in writing a consultation letter for the general practitioner and patient. Psychiatrists are blinded for the antidepressant medication (duloxetine/placebo), but not for the pain medication. Pain medication management will be provided by the psychiatrists in the duloxetine and placebo arm with collaborative care, but not in the treatment arm with duloxetine alone. In this study, care-managers and psychiatrists work according to a 'Case Registration Form' (CRF). In this CRF all the steps are described that a care-manager/psychiatrist has to do during an appointment with a patient.

#### Recruitment of patients

All the patients that are referred to the participating mental health centers are screened for depressive symptoms and pain complaints. This study will focus on patients with a depressive disorder and (sub)chronic pain complaints. Sub chronic pain is defined as pain of ≥ 6 weeks and chronic pain is defined as pain with a duration of at least 12 weeks. Consecutive patients that present themselves at the special mental health outpatient clinic will be screened for MDD and concomitant pain of ≥ 6 weeks duration with a questionnaire.

This questionnaire will consist of the PHQ-9 [13] and the item on 'average pain' from the BPI [79], that will be used as a screener for respectively depressive disorder and (sub)chronic pain symptoms. Also, the patient will receive an informed consent form with information about the study. Patients are eligible for the study if they have a score of 10 or more on the PHQ-9 and a score of 3 or more on the 'average pain' item of the BPI. When a patient screens positive on the questionnaires mentioned above, the patient will receive a telephonic interview in which a MINI interview will be administered in order to clinically confirm the diagnosis MDD and to confirm that the patient suffers from (sub)chronic pain. In that case, he or she will receive additional information about the treatment part of the study and will be asked if he or she is willing to participate. When the patient is willing to participate, the patient is included in the study. A baseline questionnaire with second informed consent form, for the treatment part of the study, will be sent to the patient’s home address or made available on a secured website. After completion of the baseline questionnaire, the patient will be randomized in one of the three interventions.

### Treatment allocation and blinding

This is a placebo controlled double blind study for the medication part of the study, which means that the allocation of duloxetine/placebo in the collaborative care arm is blinded.

Patients visiting the three participating Mental Health Institutions will be randomly assigned to one of the three treatment groups. Collaborative care and Duloxetine alone will be administered in a non-blind fashion. Administration of medication within the collaborative care conditions will be double blind and placebo controlled. Outcome assessments will be performed by a blinded research assistant.

### Deblinding

After 12 weeks, the placebo-controlled part of the study will end and the patient and physician will be deblinded for this part of the intervention. This deblinding procedure is described more detailed in the intervention.

### Emergency deblinding

Emergency deblinding of the medication condition is performed by telephone to a research associate who is not involved in treatment procedures. This happens in case of serious adverse medical events. Subjects, who are deblinded before the end of the treatment procedures, will not be incorporated in the final analysis. In case of emergency deblinding or non-compliance, the code of medication (duloxetine or placebo) in the collaborative care groups will be broken. In case of duloxetine, the medication will be reduced and stopped according to protocol. All the remaining medication of this patient will be asked back and stored in a closed cabinet, separately from the rest of the study medication. In case of non-compliance, the patient will be excluded from this study and the final analysis.

#### Patient exclusion criteria

Patients with pain for which by diagnostic medical assessment a structural and continuing physical cause has been found in terms of tissue damage, illness or otherwise, that requires treatment, such as pain due to cancer or recent post traumatic pain, are excluded from the study and advised to seek such treatment. Other exclusion criteria are:

*a PHQ-9 < 10 or a BPI score < 3,

*alcohol use >3 units (1 unit = 1 glass of ≥0.25 l) a day or drug abuse or dependence in the last 6 months, defined as current use of any hard drugs (defined by Dutch law, e.g. XTC, cocaine, heroine, magic mushrooms) or cannabis, as evident from history or, in case of suspicion during clinical interview, from laboratory findings;

*psychotic symptoms or use of antipsychotic medication that may influence perception of pain;

*use of St John’s wort (Hypericum Perforatum),

*pregnancy and breastfeeding,

*inability to participate in case of too severe language barrier,

*dementia

*history of renal and liver dysfunction for which treatment is needed

*uncontrolled hypertension despite treatment for hypertension

*Lastly, suicidal ideation is an exclusion criterion if this constitutes immediate danger and the need for crisis management according to the consulted psychiatrist. This will be measured with the suicidal ideation item of the PHQ-9. For this purpose, a suicide protocol is used in the study, defining degrees of suicide risk and prescribing necessary steps to be taken to advert such risk.

### Exclusion of the study during the intervention phase

Non-compliance is defined as not having used at least 80% of the prescribed medication (duloxetine/placebo) or no show on more than 20% of the appointments made with the psychiatrist or care-manager. The psychiatrist uses pill counts at every session to check if at least 80% of the pills have been used.

#### Intervention

##### First and second design arm: Duloxetine/placebo plus collaborative care

This intervention contains collaborative care, duloxetine or placebo, and pain medication according to an algorithm. Collaborative care will be provided within a multidisciplinary team comprising of a care-manager, psychiatrist and a physiotherapist. They all will apply treatment simultaneously according to protocol. Only patients in the collaborative care arms will receive treatment of all three specialists. The patients in the Duloxetine alone arm will only receive treatment of the psychiatrist.

**Figure 2:**
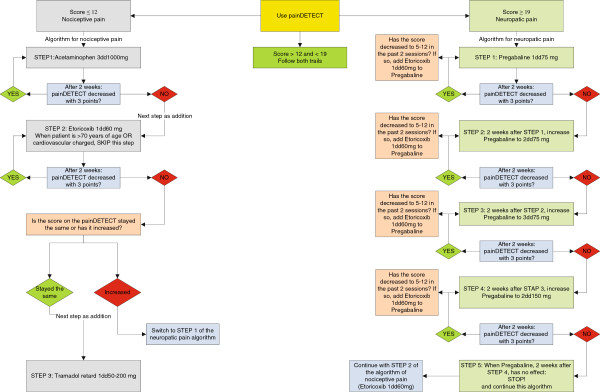
**Algorithm for pain medication.** The left side shows the algorithm for medication when the patient has nociceptive pain. The right side shows the algorithm for medication when the patient has neuropathic pain. In case of mixed pain, a switch can be done as indicated.

**Figure 3:**
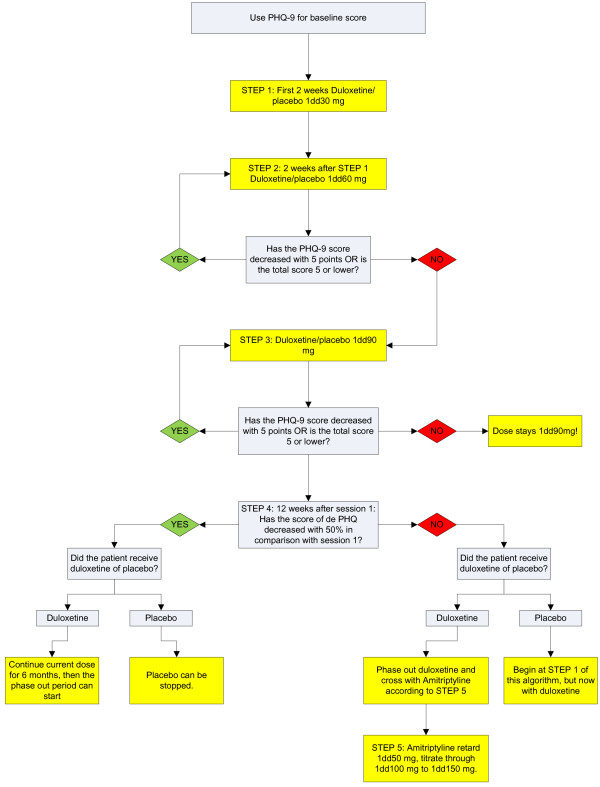
Algorithm for antidepressant medication.

The GP of the patient will be informed of the participating of the patient. Also, GP's in the same areas as the mental health centers will receive information about the study and are asked to refer patients if they have the complaints under study. The interventions will be monitored every two weeks and when needed, the doses of medication can be raised according to the algorithm shown in Figures 2 and 3. The minimum duration of the interventions will be 12 weeks. The medication blinding code will be broken after 12 weeks. A maximum of 2 sessions will follow to end the treatment. After this, medication and follow up treatment will be handed over to the GP. In case of treatment response (50% reduction on the initial score), but non-remission, as indicated by a score of > 5 on the PHQ-9 after 12 weeks, the patient will be referred back to the GP with subsequent antidepressant treatment and pain medication advice.

The detailed content of the collaborative care intervention is described below:

1. *Diagnostic assessment of the nature of pain symptoms*

The nature and the extent of the pain symptoms will be explored by the psychiatrist with the BPI and the painDETECT [74]. The 'average pain' item from the BPI will be used to assess the severity of the pain symptoms. To identify the pain symptoms as nociceptive and/or neuropathic, the painDETECT will be used. Because a patient can have both neuropathic pain and nociceptive pain at the same time, our pain medication protocol has been edited accordingly. Chronic pain may be a function of a chronic underlying pathology that appears within 4 to 6 weeks of an initial trauma; however, in accordance with the use of the terms chronic and subchronic pain in musculoskeletal pain or back pain, we apply the terms chronic and subchronic as follows: We define subchronic pain as pain with at least 6 weeks and less than 12 weeks duration, and chronic pain as pain of ≥ 12 weeks duration [6].

2. *Contracting*

During the initial visit, the care-manager informs the patient about the depressive disorder and pain symptoms, and their association. The treatment plan is then jointly formulated by the psychiatrist, the care-manager and the patient.

3. *Pain medication protocol*

In this study, pain is divided in nociceptive pain and neuropathic pain and treatment of pain will be applied based on which of these two sorts of pain a patient has. Classification as nociceptive pain, neuropathic pain or mixed pain will be done with the PainDETECT questionnaire that was specifically validated for this purpose [74].

In all instances, patients are urged to take the medication at preset intervals, in order to medicate properly so as to prevent serious pain. In all instances, duloxetine (or placebo) is prescribed as adjuvant.

The psychiatrist will use the painDETECT questionnaire to identify the pain as nociceptive or neuropathic. When it is not clear in which of these two categories the pain belongs, both the algorithms for nociceptive and for neuropathic pain are followed. The algorithm for nociceptive pain, is adapted and changed from the WHO pain ladder [80]. It is updated for the use of selective Coxinhibitors, the role of so-called adjuvants is more prominent here; the role of opioids is much less prominent, but the principle of adding medication that is common in the WHO pain ladder, is maintained. This algorithm has been developed by the research group and was tested for feasibility in patients with mental disorder in a prepilot of this trial.

Figure 2 shows a graphical version of above mentioned algorithm.

When, after 12 weeks, the patient is referred back to the GP, the GP is advised, through a Consultation Letter, to continue the medication for a duration of 6 months, as is advised in the 'NHG standard – depression' [81], a guideline for general practitioners. The GP will monitor pain symptoms with the BPI during a consult, every 6 weeks.

4. *Manual guided self-help for behavioral techniques aimed at improving coping with pain*

During the treatment, the patient works through a self-help manual, guided by the care-manager. The manual is based on several existing self-help books [43-46]. This self-help book contains information about depression and pain symptoms and their interaction, antidepressant medication, relaxation techniques and a diary for pain complaints. Every chapter contains exercises for the patients to perform. The care-manager informs the patient about the content of the manual, reinforces achievements and motivates the patient to continue. In the present study, the self-help manual is part of a complete intervention package and is therefore meant as additional to the other components of the intervention.

5. *Problem Solving Treatment*

Problem Solving Treatment (PST) is a brief, structured psychological intervention, guided by the care-manager, that has been shown to be effective in the management of depressive disorders and stress related disorders [82]. The problem-solving approach consists of 7 stages and is based on the common observation that emotional symptoms are often associated with problems in daily life and it encourages patients to formulate practical ways of dealing with such problems. The goal of PST is to teach patients to use their own skills and resources to function better [82,83], thus improving coping skills.

6. *Antidepressant medication*

In the two collaborative care conditions, patients will either receive duloxetine or placebo in a double blind fashion. The psychiatrist will monitor medication use according to protocol. In case of any adverse effects, an adverse effects protocol is followed. The pharmacists checks for possible interactions of the antidepressant medication with other medication use of the patient and instructs the doctors providing treatment accordingly.

Patients will start with a dose of 30 mg once daily in the first two weeks and 60 mg once daily from the third week. From this point the PHQ-9 will be used to measure the severity of depressive symptoms. Based on the score on the PHQ-9, the dose will be raised (when the score did not decrease; with a maximum of 90 mg) or stay the same (when the score on the PHQ-9 has decreased with 5 or more points or the score on the PHQ-9 is 5 or lower). This use of the PHQ9 for monitoring was used as well in other collaborative care studies in the Netherlands, with a focus on depression [84-87]. By using the PHQ9, comparisons can also be made with these studies. After 12 weeks the randomization code will be broken and medication will be prescribed if necessary in a standard continuation phase of at least six months. Pharmacotherapy will be gradually discontinued according to protocol and/or clinicians judgment. When the patient is referred back to the GP, the GP is advised, through a Consultation Letter, to continue duloxetine for the duration of 6 months. After this period, if possible, the GP reduces and stops the use of duloxetine in a period of 4 weeks. In case of placebo, the GP is advised to only prescribe medication when the patient still has depressive symptoms. Figure 3 shows the graphical version of the abovementioned algorithm.

7. *Monitoring treatment outcome and motivational techniques aimed at improving adherence*

Patient adherence will be improved by contracting and psycho-education and by frequent follow-up appointments in which both adherence and progress will be evaluated. Provider adherence will be improved by instructions from the researchers. Treatment outcome is monitored with a variety of questionnaires (see section 'Outcome parameters').

8. *Referral to the physiotherapist by the care-manager according to a protocol*

Next to manual guided self-help, Problem Solving Treatment and medication, patients receive physiotherapy. The physiotherapist will treat patients according to the protocol of 'graded activity'. This treatment consists of information of physical and psychological processes and how these can be intertwined. The next step is to formulate reasonable goals on what to achieve with this therapy. According to these goals, intermediate steps are formulated that have to take into account the amount of time that is predetermined.

The physiotherapist stimulates the patients with positive feedback. Also, the therapist works on a structured time schedule to optimize the course towards the determined goals. Next to the exercises, the therapist pays attention to the progress of the patients. This will be illustrated by graphs and when positive, these serve as a possible positive reinforcer and can motivate the patient.

The patients also conducts the exercises at home, so these exercises generalize to situations the patients live in. In this, the patients are stimulated by their physiotherapist.

9. *Consultation Letter*

The consultation letter is written by the psychiatrist and is meant for the GP and the patient. In this letter, the symptoms of the patient are described (somatic and psychological). This letter will also contain the treatment the patient has received in this study, as well as if the patient improved or not. The expected course of the complaints will be described, and with this an advice is given how to continue treatment of the patient by the GP. An advice is given for continuing medication (antidepressants and pain medication), the number of regularly scheduled consults with the patient every 6 weeks as checkup, to perform only physical examination if the patient presents the same symptoms again, and refrain from repeated lab or diagnostic procedures as well as to avoid hospitalization, as long as the patient does not deteriorate or presents with new symptoms. Also, the GP can consult the psychiatrist if needed. This consultation letter is sent 3 months after start of treatment, when the patient is referred back to the GP.

### Third design arm: duloxetine alone

In the Duloxetine alone condition, patients will receive duloxetine according to the algorithm as described on page 10 (*6. Antidepressant medication*). No other components, i.e. PST, manual guided self-help, pain medication, physiotherapy, and a consultation letter, of collaborative care will be given here. Patients in this condition will only be prescribed Duloxetine by a psychiatrist.

#### Data collection

Measurement will take place at baseline (T0), and three, six, nine and twelve months after baseline, respectively T1, T2, T3 and T4.

#### Outcome parameters

1. *Primary outcome measure*

#### Primary outcome parameter

Primary parameter used to substantiate the study hypothesis will be severity of depression (PHQ-9) [13]. The severity of depressive symptoms is measured with the Patient Health Questionnaire depression sub-scale [13], a brief but validated instrument that scores each of the DSM-IV criteria for major depressive disorder. Response is defined as a 50% reduction in symptoms [13,39,88]. Remission is defined as < 5 points on the PHQ-9 [88].

2. *Secondary outcome measure*

#### Secondary parameters

Secondary parameter will be the severity of pain symptoms as measured with the total BPI. The BPI has been validated for chronic non-malignant pain [79]. The localization of pain and pain being nociceptive, neuropathic or mixed of nature is assessed at the beginning of the treatment by use of the PAINDETECT questionnaire [74].

In addition to the improvement in the severity of symptoms, the cost utility of the three conditions will be compared to each other. To evaluate the cost utility of each condition, the difference in direct medical costs per patient receiving collaborative care and duloxetine, collaborative care and placebo, or Duloxetine alone is related to the difference in terms of Quality Adjusted Life Years (QALY) gained. This will yield a cost per QALY estimate. These data will be collected with the Trimbos/iMTA questionnaire for Costs associated with Psychiatric Illness (TiC-P), a measure commonly applied in economic evaluations of treatment in mental care [89,90]. The TiC-P measures direct costs of medical treatment such as the number of contacts with the general practitioner and multiple other care providers (e.g. paramedics and medical specialists) during the last three months. Medication use is measured during the last four weeks. Also, the TiC-P includes a short form of the Health and Labor questionnaire (HLQ) for collecting data on productivity losses [91], the SF-HLQ. It measures productivity loss by collecting data on absence from work, reduced efficiency at work and difficulties with job performance [92].

The EuroQoL (EQ-5D) [93] and the Short Form-36 (SF-36) [94] will be used to assess quality of life. Both are validated instruments for the measurement of general health-related quality of life. The EQ-5D measures quality of life on five dimensions, namely mobility, self-care, usual activities, pain/discomfort and anxiety depression.

3. *Additional outcome measures*

Confusion exists in terminology in describing physical symptoms and pain associated with depression [5,95]: They may be somatised symptoms of MDD, or so-called associated symptoms [96]; Medically Unexplained Symptoms, that frequently occur together with MDD [97]; chronic painful physical conditions that are symptoms of a chronic illness, such as diabetic neuropathy; or chronic painful conditions resulting from centralisation of a formerly peripheral pain symptom [63]. In this study, we use the psychiatrist's professional view on whether what kind of physical symptoms a patient has. Comorbid mental disorders will be assessed with the PHQ and GAD7 [98]. Hypochondriac tendencies will be measured using the Whitely Index [99]. The Patient-Doctor relationship Questionnaire (PDRQ-9) [100] will be used to measure the patient doctor relationship from the perspective of the patient. Physical symptoms are measured by the Physical Symptoms Questionnaire (LKV: Lichamelijke Klachten Vragenlijst) [101]. The Morisky Medication Adherence Scale (MMAS) [102] will be used to measure pain medication adherence. The questionnaire TiC-P (‘vragenlijst voor zorggebruik en productieverliezen bij psychische aandoeningen') [89] will be used to assess provided health care. To measure coping in stressful situations, the 'Coping Inventory for Stressful Situations (CISS) [103], Dutch version, will be used. To measure the tendency to overload, the 'Tendency to Overload Questionnaire' (TOQ) short form will be used [in press].

4. *Controlling variables*

The following demographic variables are measured at baseline and will be taken into account as possible effect modifiers: age, gender, nationality and ethnicity, marital status, living conditions, education and work status. Also, pain classification (neuropathic, nociceptive, subchronic, chronic) will be taken as effect modifier. Moreover, existing somatic comorbidity will be taken as effect modifier. Co-morbid chronic medical illness will be measured with the Dutch Central Bureau for Statistics list (CBS list). The list contains 28 chronic conditions (e.g. diabetes and MS).

### Sample size

#### Sample size for severity of depression as primary outcome

In a former RCT on collaborative care performed in the primary care setting, the effect size of a collaborative care intervention versus no such intervention as measured by PHQ-9 was 4.64/7.6 = 0.6 (the SD on the endpoint PHQ-9 varied between 7.0 and 8.3) [104]. In this study, we compare collaborative care plus duloxetine and collaborative care plus placebo versus the monotherapy duloxetine alone, and we compare collaborative care plus duloxetine versus collaborative care plus placebo. To have a minimum power of 80% to detect a standardized difference of 0.5 a minimum of 63 patients per arm is needed, that is 189 completers. In order to anticipate for possible loss to follow up of 20%, 3 × 79 (237) patients will be included [105].

#### Analysis

Apart from patients that will have to be excluded because of noncompliance as described above, or patients who had to undergo emergency deblinding due to serious medical adverse events, intention to treat analysis will be performed. Multi Level Analysis will be performed which will allow us to correct for variance associated with the different sites in this Multisite RCT. Effect sizes will be estimated in terms of Cohen's d and regression analysis will be performed. Possible confounders such as age, gender, immigrant status, level of education, history of treatment and life events will be included as covariates in the analysis. An independent Helmert analysis will be performed with the following contrasts: Duloxetine alone versus TCCCL + duloxetine and TCCCL + placebo; and then TCCCL + duloxetine versus TCCCL plus placebo.

To assess the cost effectiveness, we will apply a cost-utility analysis. The results will be expressed as cost per QALY. The economic evaluation will be undertaken from a societal perspective. Hence, all relevant effects and costs due to resource utilisation within the healthcare (direct medical costs) and costs due to production losses (productivity costs) will be included.

#### Controlling variables in analysis

As possible effect modifiers, co morbid mental health disorders as measured with the PHQ (e.g. anxiety) will be taken into account. Another parameter will be the severity of hypochondriac tendencies (WI) as a possible effect modifier [99]. The CBS list ‘chronic diseases’, developed by the Dutch Central Bureau of Statistics, will be used to evaluate physical co morbidity that may cause pain (e.g. Diabetes, Multiple Sclerosis). Process measures will be compliance and adherence to treatment, measured by the self-report questionnaire Morisky Medication Adherence Scale (MMAS) [102] and the patient-doctor relationship as measured by the PDRQ-9 [100], as well as assessment of the care provided in both conditions [89]; this includes care the patients have received up to a year before they fill in the baseline questionnaire.

#### Timeframe of the study

The goal is to enroll 219 patients in order to obtain 189 patients that will complete the study. The preparatory period is approximately 6 months. Care-managers will be recruited subsequent to the approval of the Medical Ethical Board and the care-managers will be trained. The inclusion and intervention period will be 24 months. The follow-up phase will last 12 months. Data analysis will take 6 months. The entire study period is 4 years.

#### Ethical principles

This study will be conducted in accordance with the code of ethics that has been established by the declaration of Helsinki (1964) and amended in Edinburgh (2000). Subjects will be informed of all procedures and asked for written informed consent. The patients will be informed that they can withdraw their consent to participate at any time without specification of reasons and without negative consequences with regard to future medical treatment. A medical adverse events protocol is established. Every session with the psychiatrist side-effects, that may have occurred since the last session, will be measured with the Dutch translation of the Antidepressant Side-Effects Checklist (ASEC-21) [106]. When the patient has a serious side-effect not mentioned on the ASEC-21, an adverse medical events protocol will be followed, involving emergency deblinding.

The study has been approved by the Medical Ethics Committee of the VU Medical Centre (reference number: 08072).

### Dissemination of results

This study will establish cost effectiveness of TCCCL and then make the following products available for long term implementation:

– Training module of psychiatrists and psychiatric nurses working in a specialty mental health outpatient clinic to perform TCCCL integrated depression and pain treatment, and to refer the patient back to the GP with a CL letter within 3 months.

– Format of a Consultation Letter to the GP.

– Algorithm for psychiatrists about how to monitor and improve the pain medication of the patient according to a protocol.

– A manual for the psychiatric nurses to monitor progress and to use the DVD ‘Facts on Depression’ in psycho education for the patient.

– A self management manual for patients including activation and relaxation techniques.

– A practical protocol for duloxetine use in patients with depression and pain.

## Discussion

This paper describes the study protocol of a Multi-Center randomized controlled trial evaluating collaborative care, antidepressant medication and pain medication in the treatment of depressive disorders with (sub)chronic pain. The aim of this study is to compare three treatments in terms of (cost)effectiveness.

A strength of this study is that the three arm design enables us to show the value of a closely monitored integrated treatment model above usual pharmacological treatment. Another strength of the study is that the comparison with a placebo arm enables us to evaluate effectiveness of duloxetine in this population in a real life setting.

Also, a strong aspect of this design is that this will provide evidence-based treatments and tools for their implementation in practice. This will facilitate generalization and implementation of results of this study.

Furthermore, patients included in this study are screened for pain symptoms, differentiating between nociceptive and neuropathic pain, unlike the studies of Goldstein et al. [107]. Therefore, pain relief can be thoroughly evaluated.

Another strength of the study is the structured implementation of a transmural model, aimed at better collaboration between GP and specialty mental health clinic in complex patients with depression and concomitant (sub)chronic pain.

A limitation of the study is that, with this study design, we will not be able to make inferences about the effectiveness of the respective ingredients of the collaborative care model (such as PST or the self-help manual).

## Abbreviations

TCCCL: Transmural collaborative care with consultation letter; CC: Collaborative care; MDD: Major depressive disorder; GP: General practitioner; PST: Problem solving treatment; CL: Consultation letter.

## Competing interests

In the past three years CFC received royalties for books that she wrote on psychiatry and her employer (EdH) received a research grant for this Investigator Initiated Trial (IIT) from Eli Lilly. JD: his employer (FK) received a research grant for this IIT from Eli Lilly. The other authors have no competing interest.

## Authors’ contributions

CFC is the principle investigator. She supervises and facilitates EdH, facilitates data management and data handling and statistical analysis in the study. She designed this study and acquired funding for the study. She developed the algorithms for duloxetine, and for the pain medication. She recruited physiotherapists for the study, took part in the training for psychiatrists and is available for consultation by the participating psychiatrists. She facilitated and coordinates the study at GGz Breburg. She co-authored this article. EdH participated in the study design, conducts the trial, assisted in the training and supervision of the care-managers and psychiatrist, recruited physiotherapists for the study and wrote this article. JD participated in the design and acquisition of funding of this study, facilitated the study at Arkin and co-authored this article. HvM participated in acquisition of funding of this study, supervised the PST training and co-authored this article. AB facilitated the study in GGz inGeest and co-authored this article. JR participates in the trial and coordinates the trial at GGz inGeest and coauthored this article. TH participated in implementing the start of the study in the outpatient clinic of Arkin mental health care, Amsterdam and co-authored this article. PB checked all medication for interactions and coordinated the distribution, randomization and blinding of the antidepressant medication. JvEvdS tested the medication algorithms, assisted in recruiting physiotherapists in GGz Breburg and co-authored this article. LH contributed to the design of the economic evaluation of this study and coauthored this article. FK participated in conducting this trial in Arkin and assisted in the supervision of the care-managers and psychiatrist and coauthored this article. LR co-authored this article. All participants contributed their own specific expertise and read and approved the final manuscript.

## Pre-publication history

The pre-publication history for this paper can be accessed here:

http://www.biomedcentral.com/1471-244X/13/147/prepub
